# Rattlesnakes and Delirium Tremens

**Published:** 1854-10

**Authors:** N. Gilman

**Affiliations:** South Deerfield


### RATTLESNAKES AND DELIRIUM TREMENS.

[From the Boston Medical and Surgical Journal.]

Messrs. Editors:—As I have never been in a region infected with poisonous serpents, I have no practical knowedge of the best mode of treating their bite. If alcoholic liquors are a specific in such cases, the thing is entirely new to me. This, however, seems to be taken for granted by Dr. Hall, and another correspondent of the Journal in Philadelphia who writes over the signature B. As controversy is no part of my object, a desire for truth alone influencing me in my present course, these writers will pardon me if I lay them under a slight contribution. I wish to learn 1st, How long it has been known to the medical profession, that alcohol is a specific for the bite of the rattlesnake? 2d, Is the bite of the reptile always fatal under ordinary treatment, or no treatment at all? 3d, Does the alcoholic treatment ever fail? 4th, What form of alcohol is best, and what the best mode of exhibition?

I confess that I have serious doubts, in my own mind, whether alcohol is entitled to any credit in this connection. In the cases which I have seen reported, there were other powerful medicines used at the same time—in one case the patient taking twenty grains of carb. ammon. every half hour alternately with a glass of whisky; in another, taking large quantities of spirits through the night, without any relief, but in the morning adding large doses of opium and morphia. Under this treatment the patient experienced immediate relief, and soon recovered. Yet rum gets the whole credit. In communities where they love rum, there seems to be a tacit understanding that this vile article shall have the credit of curing every person that recovers from disease of any kind, while calomel and the lancet must bear the blame of killing all who die.

I understand Dr. Hall to express the opinion that intoxicating liquors should be kept for the bites of snakes alone, even if valueless for any other purpose. This brings us to the great objection—the danger, or with more propriety we might say, the certainty, that their medicinal use will make drunkards. This will be the subject of my next article. But the rattlesnake argument would not affect us here in Massachusetts. Not being much troubled with the poisonous snakes, we do not need the antidote. We have men enough among us, however, who would import and breed them, if no other excuse could be found for the rum traffic. I think the Doctor, who expresses himself very strongly against the use of spirits as a beverage, would find it difficult to confine them to this particular use. To secure the full benefit of the remedy, it should be used immediately after the bite. Hence in the Western States it would be necessary to keep spirits in every family, and no person could prudently venture into the fields or woods, half a mile from home, without a bottle in his pocket; though, as a matter of course, he would not drink any unless he was bitten, or feared that he should be. I have seen one case reported, in which a man was bitten while in a state of intoxication, without producing any effect. How it affected the snake, does not appear in the report, although rumor says that he died. This would afford quite an argument in favor of their use as a preventive. I would like to enquire of Dr. Hall and Dr. B. whether, in the course of their rattlesnake practice, a reformed inebriate has ever come under their care; and if so, if his old dormant appetite was aroused by the copious whisky potations? In the Journal of April 6th, 1853, is a case taken from a southern Journal, of a very peculiar character. A young lady was bitten by a rattlesnake, treated with whisky and carb. ammon. to the extent of three pints of the former and eighty grains of the latter, and cured. "But the most interesting feature of the case remains to be stated. The lady, at the time she was bitten, was the subject of a well-marked hooping cough. The disease was of about three weeks standing, consequently it was at its acme. On recovering from the effects of the poison, to her surprise and gratification the cough was gone, and never returned. Being essentially a nervous affection, it was swept away at once by the powerful impression upon the nervous system." What would the Doctor think of the plan of keeping rattlesnakes to cure the hooping cough?

But "what would Dr. Gilman use?" For eight years past it has been known to the profession in Illinois, that iodine is a valuable remedy for the bite of the rattlesnake, and successful cases have been reported to the N. W. M. and S. Journal, by two physicians, at least. For some time, also, it has been known in that region, that the simple affusion of cold water upon the parts was an effectual remedy. Several well-authenticated cases of this kind have occured within a year, and been published in the Water-Cure Journal. Should such a case come under my care, I should combine the two modes of treatment; using the iodine externally and internally, to counteract the poison, and at the same time pouring ice water on the swollen part.

In delirium tremens it is really lamentable that the profession still hold on to such an unscientific and barbarous mode of practice as the rum treatment. Were spirituous liquors the only remedy for this disease, this fact would afford no argument against abandoning the use of alcohol, as the *cause* would thereby be removed. But there is a better way. It is this. Let the patient have two or three buckets full of cold water poured on his head once in a few hours, taking large doses of morphine every half hour, till sleep is produced. This plan of treatment is much more successful than the "the-hair-of-the-same-dog" treatment. And, what is of vastly more importance still, the patient is put into a better condition for reformation; while by the other, he is only forced on another step down towards perdition. I have recently tried this plan on a young man in my own neighborhood, with the most perfect success. This patient was what is termed a "hard case." Although but twenty-four years of age, he had been a drunkard for eight years. He had delirium tremens twice before, and had become possessed with the idea that he should die if he left off drinking. For six or eight months he had averaged one and a half quarts of cider brandy in a day. By taking this course with him, he was made to see the fallacy of his own opinions, and induced to make an effort to reform. After a few weeks' abstinence, satisfying himself that his life would not be endangered, he signed a pledge never to use any alcohol, in any form or combination, for any purpose whatever, to the end of his life. This pledge he has kept for six months, has gone into business, and bids fair to be a useful man. Had he been saved by the rum treatment, he would have remained the same degraded, worse than useless being—not cured, but merely respited for a short time from his awful doom, and the system put into a more complete state of preparation for a recurrence of the disease. I am decidedly of the opinion that a physician does no good, either to the patient, friends, or to the community generally, by curing a case of delirium tremens, unless he reforms the man. Then let him not, in the outset, resort to those means which will preclude the possibility of his reformation.

There are some things, besides the rattlesnake bite, in B.'s article, which will be noticed another time.

South Deerfield, *June 8th*, 1854. N. Gilman, M. D.

